# Supplementary material for: Standardization of laparoscopic trays using an inventory optimization model to produce immediate cost savings and efficiency gains
Source: PLoS One. 2022 Dec 29;17(12):e0276377. doi: 10.1371/journal.pone.0276377 (PMC9799292; doi:10.1371/journal.pone.0276377)
Supplement: S1 File — (DOCX) [file pone.0276377.s001.docx]

# **Supporting Documentation**

**S1 Table. General Surgery laparoscopic tray.**

| Picture of Instrument | Instrument Name | Quantity |
| --- | --- | --- |
| 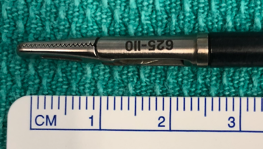  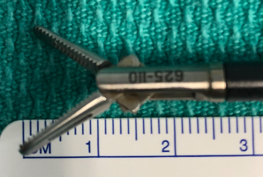 | Dissector | 1 |
| 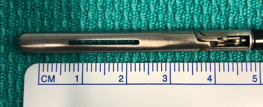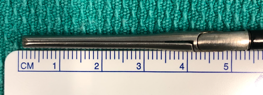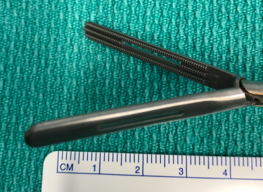 | Debakey flat fenestrated dissector | 2 |
| 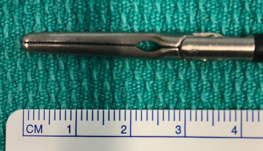  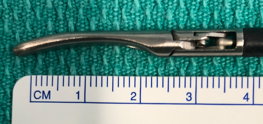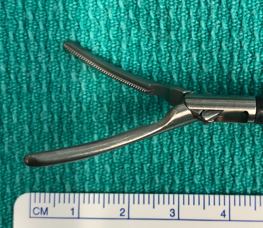 | Crile | 1 |
| 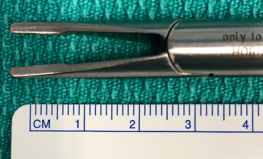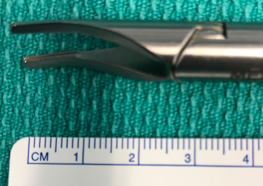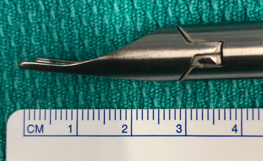 | Horizontal Clip Applier (Med Green) | 1 |
| 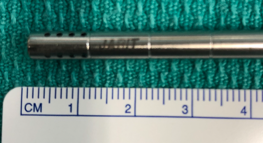  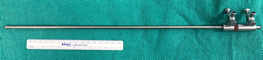 | Suction Irrigator | 4 |
| 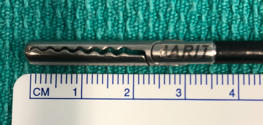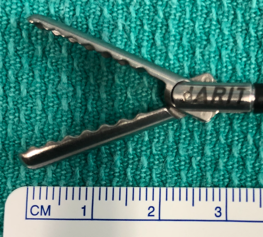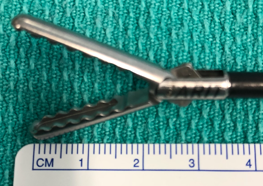 | Fundus Grasper/ Bowel Grasper/ Wavy Recessed Atraumatic Grasper | 3 |
| 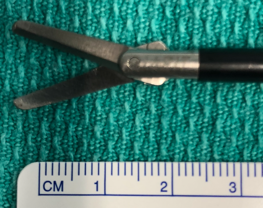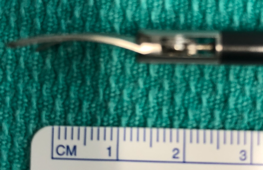 | Lap Scissors (Endoshears) | 1 |
| 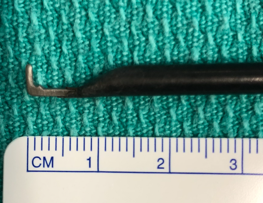 | L Hook | 1 |
| 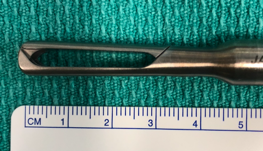  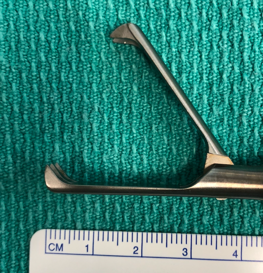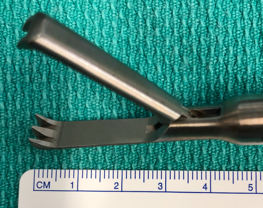 | Claw Forceps | 1 |
| 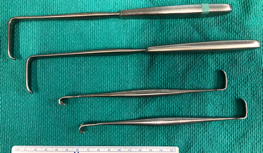 | Senn Retractors | 2 |
| 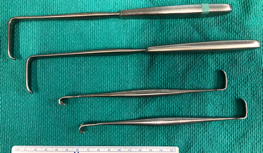 | Right angle retractor | 2 |
| 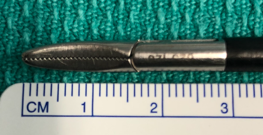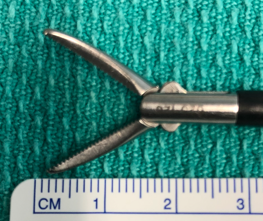 | Maryland Dissector | 1 |

**S2 Table. Gynecology laparoscopic tray.**

| Picture of Instrument | Instrument Name | Quantity |
| --- | --- | --- |
| 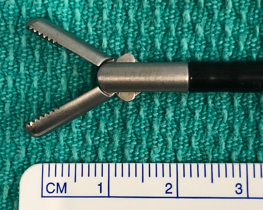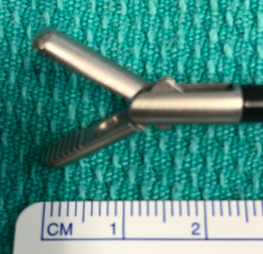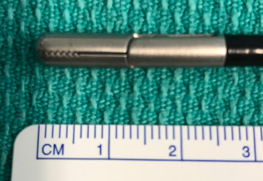 | Round Grasper/ Bullet Grasper | 2 |
| 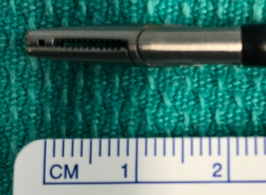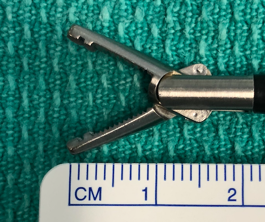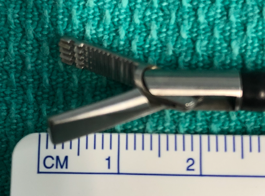 | Fundus Grasper/ Atraumatic Allis Grasper | 1 |
| 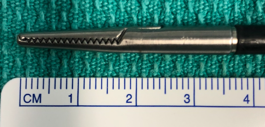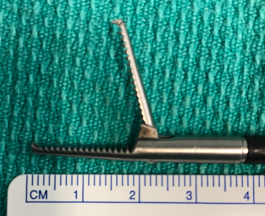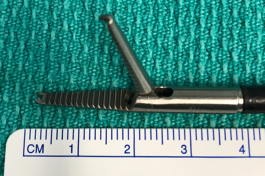 | Needle-Nosed Toothed Grasper | 1 |
| 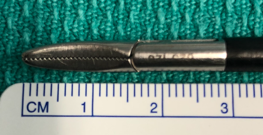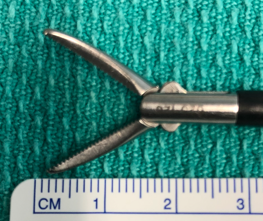 | Maryland Dissector | 1 |
| 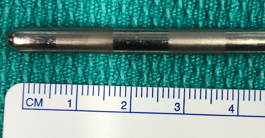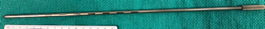 | Probe | 1 |
| 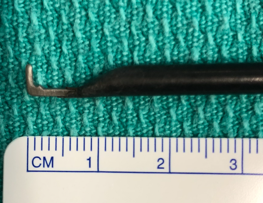 | L Hook | 1 |
| 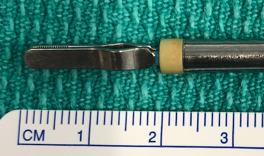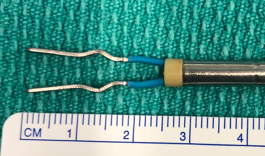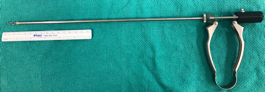 | Bipolar forcep | 1 |
| 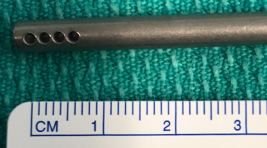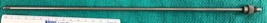 | Suction irrigator (Stryker) | 1 |
| 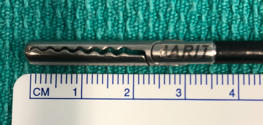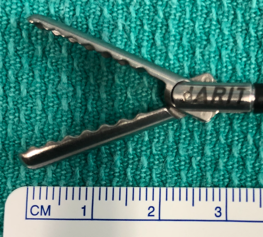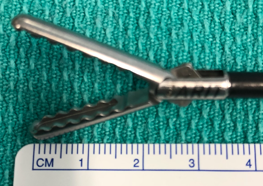 | Fundus Grasper/ Bowel Grasper/ Wavy Recessed Atraumatic Grasper | 3 |

**S3 Table. Gynecology oncology laparoscopic tray.**

| Picture of Instrument | Instrument Name | Quantity |
| --- | --- | --- |
| 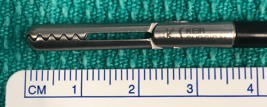  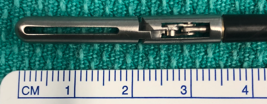  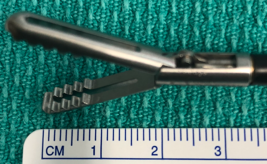 | Lap Clinch/ Fenestrated Atraumatic Wavy Grasper | 2 |
| 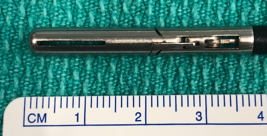  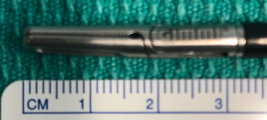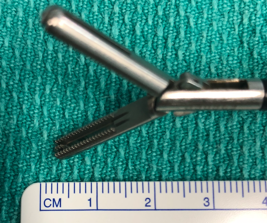 | Hunter Grasper/ Debakey Tip Recessed Grasper | 2 |
| 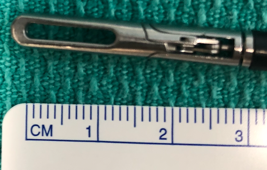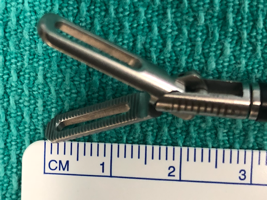 | Duckbill Grasping Forcep | 2 |
| 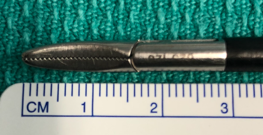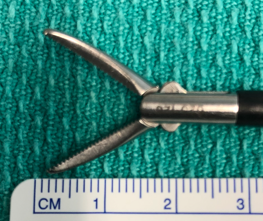 | Maryland Dissector | 1 |
| 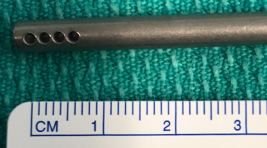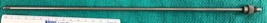 | Suction irrigator (Stryker) | 1 |
| 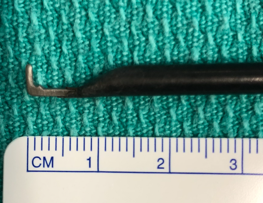 | L Hook | 1 |
| 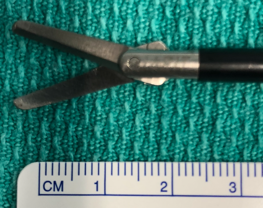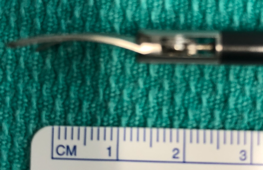 | Lap Scissors (Endoshears) | 1 |
| 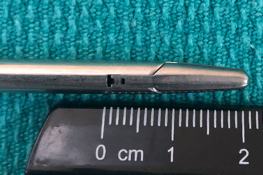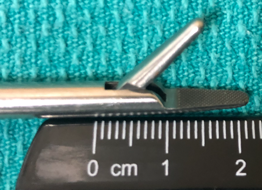 | Needle driver | 1 |

**S4 Table. Standardized laparoscopic tray.**

| Picture of Instrument | Instrument Name | Quantity |
| --- | --- | --- |
| 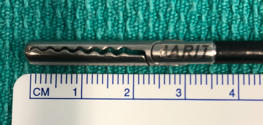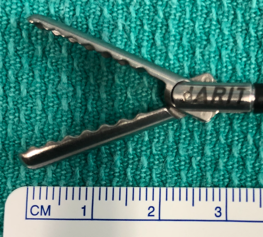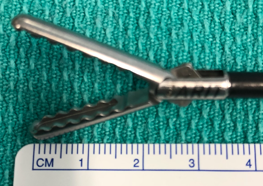 | Bowel Grasper/ Wavy Recessed Atraumatic Grasper  (2 ratchet, 1 non-ratchet) | 3 |
| 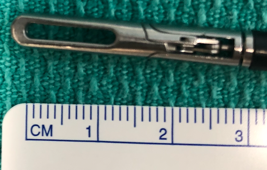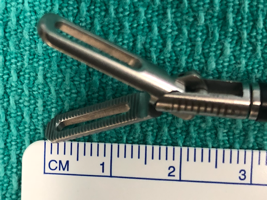 | Duckbill Grasping Forcep | 2 |
| 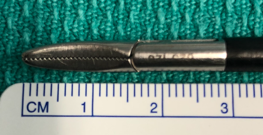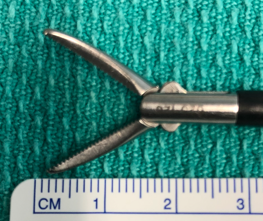 | Maryland Dissector | 2 |
| 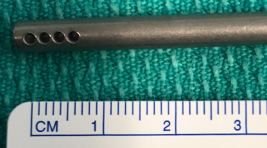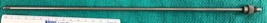 | Suction irrigator (Stryker) | 1 |
| 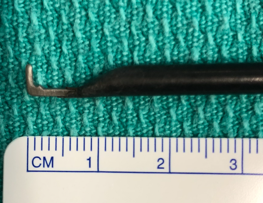 | L Hook | 1 |
| 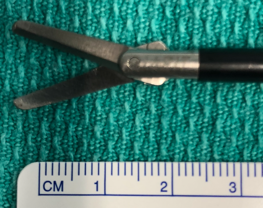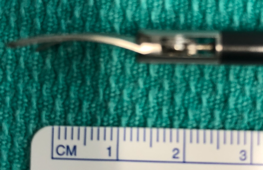 | Lap Scissors (Endoshears) | 1 |
| 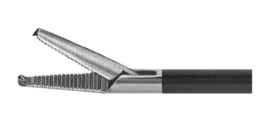 | Lap Kocher | 1 |

**S5 Equation.**  The $z$-score.

The $z$-score may then be derived as follows:

$$z={F^{-1}\left( CSL \right)}$$

, $F^{-1}(x)$is the inverse normal distribution function. Using an assumption of 99% service level (as shown in Equation 4) simplifies the $z$-score to a constant.
