# Supplementary material for: Standardization of laparoscopic trays using an inventory optimization model to produce immediate cost savings and efficiency gains
Source: PLoS One. 2022 Dec 29;17(12):e0276377. doi: 10.1371/journal.pone.0276377 (PMC9799292; doi:10.1371/journal.pone.0276377)
Supplement: Attachment — Submitted filename: Lap Tray Standardization Response to Reviewers.docx [file pone.0276377.s003.docx]

| **Dr. Federico Ferrari**  Academic Editor  PLOS ONE  February 16^th^, 2022 | | **Dr. Jay Toor**  Department of Surgery  University of Toronto  Toronto, ON, Canada |
| --- | --- | --- |
|  |  | |

Dear Drs. Ferrari and Chenette,

Thank you on behalf of the reviewers for your consideration of our manuscript “*Standardization of laparoscopic trays using an inventory optimization model to produce immediate cost savings and efficiency gains*” to be considered for publication in *PLOS ONE.* We have taken the time to consider the feedback from the reviewers, and we have made the following changes to the manuscript:

Reviewer #1:

“*First, the statistical method is not very clear in this article”* Thank you kindly for this comment. We elaborated that we used a side-by-side cost comparison (Line 149-150). We have also described our methodology in detail including equations. Having incorporated the suggestion, we are confident the readers of PLOS ONE will be able to better understand our work.

“*The actual clinical application effect in different regions needs to be further analyzed by increasing the number of samples and adopting multi-center method*” Thank you for this insightful comment. We included the suggestion in Line 266-*267*.

Reviewer #2:

“There are some grammatical and typing errors in this manuscript.” Thank you for this constructive feedback. We have reviewed the manuscript in great detail and corrected any grammar and punctuation errors (Line 50, 75).

Thank you for your time and efforts in enhancing our work.

Sincerely,

Jay Toor MD MBA

Orthopaedic Surgery Resident Physician

Department of Surgery

University of Toronto

Tel: 1-416-918-9519

Email: jaysinghtoor@gmail.com
