# Supplementary material for: Standardization of laparoscopic trays using an inventory optimization model to produce immediate cost savings and efficiency gains
Source: PLoS One. 2022 Dec 29;17(12):e0276377. doi: 10.1371/journal.pone.0276377 (PMC9799292; doi:10.1371/journal.pone.0276377)
Supplement: Attachment — Submitted filename: comments.docx [file pone.0276377.s004.docx]

This manuscript is well revised and could be accepted.
